# Predicting the membrane permeability of organic fluorescent probes by the deep neural network based lipophilicity descriptor DeepFl-LogP

**DOI:** 10.1038/s41598-021-86460-3

**Published:** 2021-03-26

**Authors:** Kareem Soliman, Florian Grimm, Christian A. Wurm, Alexander Egner

**Affiliations:** 1 Institute for Nanophotonics Göttingen e.V., Optical Nanoscopy, Hans-Adolf-Krebs Weg 1, 37077 Göttingen, Germany; 2 Abberior GmbH, Hans-Adolf-Krebs Weg 1, 37077 Göttingen, Germany; 3 Faculty of Physics, George-August University, Göttingen, Germany

**Keywords:** Cheminformatics, Imaging techniques

## Abstract

Light microscopy has become an indispensable tool for the life sciences, as it enables the rapid acquisition of three-dimensional images from the interior of living cells/tissues. Over the last decades, super-resolution light microscopy techniques have been developed, which allow a resolution up to an order of magnitude higher than that of conventional light microscopy. Those techniques require labelling of cellular structures with fluorescent probes exhibiting specific properties, which are supplied from outside and therefore have to surpass cell membranes. Currently, major efforts are undertaken to develop probes which can surpass cell membranes and exhibit the photophysical properties required for super-resolution imaging. However, the process of probe development is still based on a tedious and time consuming manual screening. An accurate computer based model that enables the prediction of the cell permeability based on their chemical structure would therefore be an invaluable asset for the development of fluorescent probes. Unfortunately, current models, which are based on multiple molecular descriptors, are not well suited for this task as they require high effort in the usage and exhibit moderate accuracy in their prediction. Here, we present a novel fragment based lipophilicity descriptor DeepFL-LogP, which was developed on the basis of a deep neural network. DeepFL-LogP exhibits excellent correlation with the experimental partition coefficient reference data (R2 = 0.892 and MSE = 0.359) of drug-like substances. Further a simple threshold permeability model on the basis of this descriptor allows to categorize the permeability of fluorescent probes with 96% accuracy. This novel descriptor is expected to largely simplify and speed up the development process for novel cell permeable fluorophores.

## Introduction

**Figure 1:**
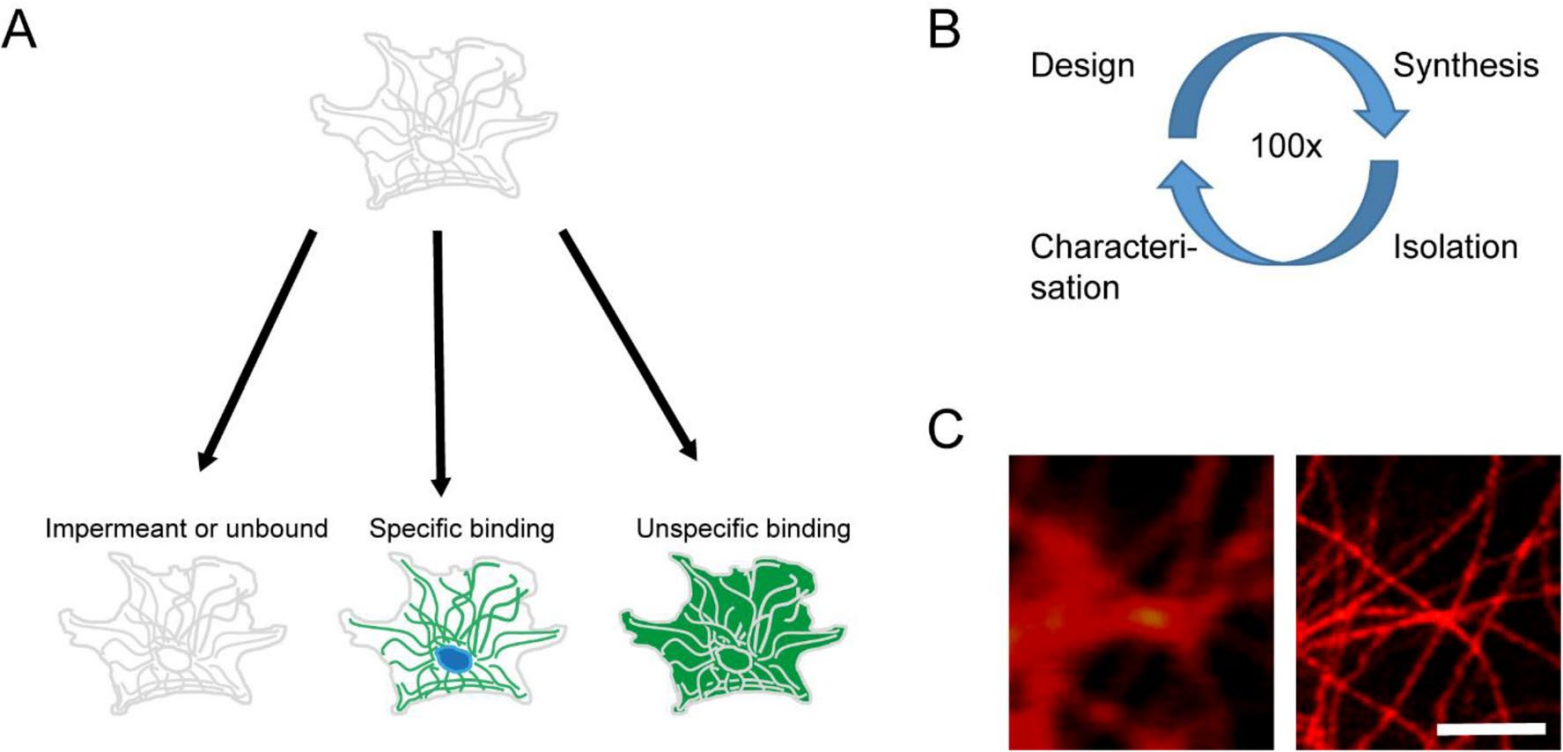
Labeling of living cells and screening for cell permeable fluorescent probes. (**A**) Labelling of living cells with fluorescent probes can result in three scenarios: Impermeant fluorescent probes or probes that fail to bind to a target molecule resulting in an unstained cell (left), cell-permeable fluorescent probes which exhibit specific binding result in good staining (middle), permeable fluorescent probes which exhibit unspecific binding result in undefined staining patterns (right). (**B**) Classical screening approach to develop new probes. (**C**) Exemplary confocal and STED image of the tubulin cytoskeleton in living cells. Scale bar, 1 µm.

Small-molecule fluorescent chemical probes are important tools for bioimaging applications. The recent advances in super-resolution nanoscopy enable scientists to routinely image biological samples with a resolution down to few nanometers^1–3^. These, so called, nanoscopes are also integrated into fully automated platforms, which is important for high-throughput screening (HTS) applications in drug discovery and toxicity research^4^. The direct visualization of intracellular targets in-vivo*/*in-vitro at this unprecedented resolution requires the use of fluorescent probes with excellent cell permeability, high specificity and low background (Fig. 1A).

Identification of cell permeable probes within a large set of available regular fluorophores is nowadays still based on a trial and error approach that involves screening hundreds of compounds (Fig. 1B), as the final probe should exhibit excellent cell permeability and specific binding to cellular targets (Fig. 1C), HTS synthesis platforms can speed up this process but are tedious and costly.

Therefore, the development of new methods for prioritizing chemical designs is an attractive alternative. **Q**uantitative **S**tructure **A**ctivity **R**elationship/**Q**uantitative **S**tructure **P**roperty **R**elationship (QSAR/QSPR) models predict the activity/property of potential probes on the basis of molecular descriptors and are increasingly used as prioritization tools for drug/probe development^5–7^. The accuracy of the algorithms used to calculate these descriptors is crucial for the reliability of these models, and hence also for the precision of the prioritization tools.

When it comes to cell permeability the LogP descriptor is the most significant descriptor^8–11^. It has been shown that drugs with good cell permeability exhibit moderate LogP values^12,13^. The Lipinski rule of five that is used to evaluate the drug-likeness of compounds indicates a moderate LogP range (− 0.5 < LogP < 5) for substances with good cell permeability^14^. Various LogP descriptors have already been developed that can be used to build models for cell permeability.

Early algorithms calculated the LogP purely on the contribution of single atoms^15^. Enhanced/hybrid atomistic algorithms (SLogP/XLOGP3/MLOGP) have been developed to overcome some of the shortcomings of the atomic algorithms and take also the contribution of neighboring atoms and hybridization into account^16,17^. Fragment LogP descriptors are based on a different approach. They use the experimentally determined partition coefficient of chemical fragments or compounds as a basis for a QSPR model or a regression technique which then predicts LogP^18^. Fragment descriptors (e.g. miLogP, Molinspiration) take therefore the nuances of electronic or intramolecular interactions into account, which is not the case for atomistic algorithms. Consequently, they tend to perform better for larger molecules or compounds with more complex chemical structures, like fluorescent chemical probes. Another class of algorithms, e.g. MLogP^19^, calculate LogP by using molecular properties such as 3-D structures or topological indices. The consensus LogP (cLogP) descriptor calculates the average LogP from multiple LogP models with improved accuracy^20^. In addition to these LogP models based on chemical properties, there exist also physics based algorithms which estimate the LogP from the computed solvation free energy of organic compounds in implicit media, such as the iLOGP descriptor^21^. Finally, the rapid development of machine learning, e.g. artificial neural networks, has increased the accuracy and speed of many of these LogP descriptors^22^.

Recently, LogP has been used for the first time to build a QSPR model for the development of cell permeant fluorescent probes^5^. In this study it has also been shown that cell-permeable fluorescent molecules tend to exhibit LogP values greater or equal to 1 and that this can be used as a threshold in the design criteria of cell permeant dyes. However, it has not been quantitatively tested whether the LogP descriptor could be solely used to accurately categorize the permeability of this molecule class.

To address this important question, we screened several LogP descriptors by analyzing a multitude of more than 100 permeant and impermeant probes using various LogP algorithms and a predefined LogP threshold. Furthermore, we increased the accuracy of the algorithm by introducing a new deep neural network based LogP descriptor (DeepFL-LogP) that can categorize the permeability of fluorescent probes with superior accuracy.

## Materials and methods

### LogP calculations

SLogP has been calculated using the Mordred Python package^23^. XLOGP3, MLOGP, cLogP, and the iLOGP have been calculated using the SwissADME web tool^20^. After calculation, all descriptors were merged into one file for subsequent statistical analysis.

### Test dataset of fluorescent probes

In total 124 fluorescent probes and fluorophores were used for evaluation (Table S1). A diverse and a wide range of types of fluorophores is covered (Fig. S1). The structural formula of the majority of the fluorescent probes were found along with their Simplified Molecular Input LINE Entry (SMILES) codes in^24^. Corresponding information on additional fluorescent probes was extracted either from literature or commercial catalogues. In cases where the SMILES codes were not accessible, the chemical structures were used to generate SMILES codes.

Information on probes’ permeability were manually curated from commercial catalogues or literature. In case of literature search, individual publications were manually explored for permeability data. Only when a fluorescent probe has been applied to live cells and there was clear microscopic evidence about its cellular localization^25^, it was considered to be a cell-permeant probe. A probe was considered to be impermeant if it preferentially stains fixed or dead samples (e.g. Propidium iodide and Evans Blue)^25,26^.

### Deep neural network architecture and training

The deep neural network (DNN) has been developed using the Keras library in Python^27^. The architecture of the neural network is based on a simple sequential model that consists of three main sequentially connected layers: An Input layer, three hidden layers, and an output layer. The input layer consists of 319 neurons, which corresponds exactly to the number of molecular features used. A sigmoid activation function has been added to this layer. The network’s three hidden layers consist of: a first layer of 256 neurons (sigmoid activation function), followed by a second layer of 164 neurons (tanh activation function) and a third layer of 10 neurons (sigmoid activation function). The output layer consists of a single neuron with a single output. Since this is a linear regression-like problem and the network is purposed to predict LogP values based on experimental determined values (i.e. logarithm of measured partition coefficients), an activation function was not added to the final output neuron layer and raw values were directly used.

Hyperparameter tuning in neural network-based models is essential for accurate predictions^28^. For error backpropagation and error minimization during the training process of the neural network, a Stochastic Gradient Descent (SGD) optimizer function with a learning rate and nesterov momentum of 0.01 and 0.9, respectively, has been implemented. For loss monitoring during the training process, the root mean square function was calculated for each epoch (i.e. an epoch corresponds to one learning cycle using the entire training set). A total of 78 epochs was found to be optimal for the pre-defined learning rate.

The batch size controls the size of samples to be used to estimate the error gradient before the model weights are updated and is therefore another important hyperparameter for DNNs. In this study a batch size of 32 was used. To improve the networks performance and to reduce bias, samples were randomly shuffled prior to the start of the training process. Training was performed in Colab (https://colab.research.google.com) utilizing the GPU in order to speed up the process. The final trained model architecture and weights were stored in two separate HDF5 files.

### Fingerprints and chemical fragment analysis

The 2D RDKit Electronic State (E-state) fingerprint algorithm was used to determine the atom types as well as the basic fragment descriptors for each molecule^29^ (Table S2). Descriptors of supplementary basic as well as larger/complex fragments, which are not provided by the RDKit, were additionally calculated using functions provided by the kit (Table S3). Overall 319 features per molecule were used to train the final model.

### Training/validation datasets

Experimental lipophilicity LogP values of more than 13,000 drug-like molecules from the curated and publicly available OPERA dataset were used to train and validate the neural network model^30^. In order to expand the chemical space and to improve the model’s accuracy for fluorescent probes, information on 222 auxiliary molecules together with their experimental LogP or in case of ionizable compounds LogD values were added to the OPERA training set (Table S4). Only twenty four compounds of the total are fluorescent (Fig. S1). If any of those additional molecules was already contained in the OPERA-validation set, it was removed therefrom. Overall, the training and validation data sets used consist of 10,749 and 3502 molecules respectively.

### Data and statistical analysis

(Statistical) analysis of the data was performed in Python using the Spyder IDE. The histogram-based analysis of the experimental LogP data and the distribution (boxplot) analysis were performed using the seaborn library. Descriptive statistical measures (mean, min, and max) of the training and test sets were calculated using basic Python functions. The regression coefficient (R^2^) and the mean square error (MSE) were calculated using built-in functions of the Scikit-learn library^31^. Pearson and t-test analysis were performed using the SciPy statistics library. To determine statistical significance for the LogP analysis, two-independent sample t-tests with unequal variances were calculated.

### Stimulated Emission Depletion (STED) microscopy

For the labeling of the tubulin network of living U-2 OS cells, the cells were seeded on coverslips one day before the experiment as described before^32^. Labelling was performed for 1 h with a 2 µM staining solution of Abberior LIVE 610-Tubulin (λ_exc_ 609 nm, λ_em_ 635 nm) in cell culture medium under cell culture conditions. Imaging was performed on an Abberior Instruments Facility Line Microscope using a pulsed excitation laser at 561 nm and detection window between 570 and 680 nm. STED images were recorded using a STED laser at 775 nm.

## Results

### LogP and permeability

Previous permeability models have emphasized that a LogP threshold value (LogP ≥ 1) is adequate to distinguish permeant from impermeant compounds, the latter exhibiting lower LogP values^5,8^. Using this threshold several descriptors were tested in order to determine how accurately the permeability of probes can be categorized. For this purpose the LogP values for a test set containing 124 fluorescent probes of known membrane crossing profile were calculated with the six descriptors investigated here and categorized according to the threshold value of 1. The set consists of n = 99 permeant with diverse subcellular localizations and n = 25 impermeant probes (Table S1).

**Figure 2:**
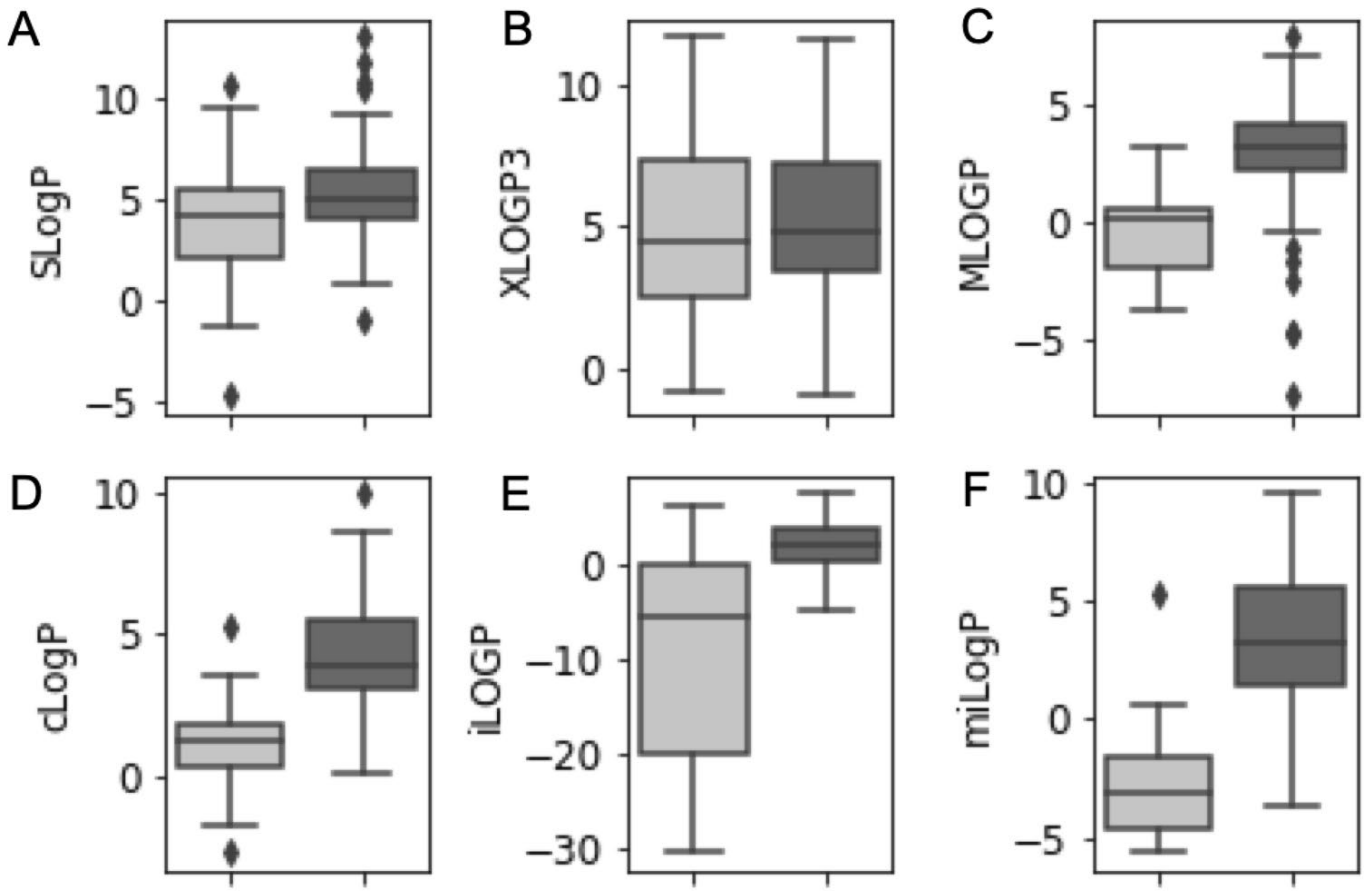
Distribution of LogP values for a test set of fluorescent probes. The boxplots display the distribution of six LogP descriptors calculated for a set of 124 fluorescent probes (25 impermeant probes, light grey and 99 cell permeable probes, dark grey). (**A**) SLogP descriptor. (**B**) XLOGP3 descriptor. (**C**) MLOGP descriptor. (**D**) cLogP descriptor. (**E**) iLOGP descriptor. (**F**) Boxplot of the miLogP descriptor.

The analysis shows that the atomic descriptors (SLogP/XLOGP3) display high LogP values regardless of the probes’ permeability (Fig. 2A,B). In detail, impermeant as well as permeant probes show positive SLogP values with a majority (98%) of permeant and (88%) of impermeant probes exhibiting values equal or greater than 1. The majority (91%) of permeant and (88%) of impermeant probes exhibit also a XLOGP3 that is equal or greater than 1. On the other hand, the atomic MLOGP descriptor shows a significant higher LogP average in case of permeant probes in comparison to impermeant probes (*p* value = 1.2e−08, Fig. 2C). The majority (88%) of the permeant probes exhibited a MLOGP value equal or greater than 1, while the majority (76%) of the impermeant probes exhibited lower MLOGP values. The consensus LogP (cLogP) descriptor, which is the arithmetic mean of some of the best LogP models^33^, exhibited a similar difference between the permeant and impermeant probes (*p* value = 4.5e−10). The majority (98%) of permeant probes showed a value equal or greater than 1 (Fig. 2D). However, only 36% of impermeant probes exhibited low cLogP values (less than 1). The recently developed physics based LogP descriptor (iLOGP) revealed a majority (70%) of permeant probes to have an iLOGP equal or greater than 1, while the majority (80%) of impermeant probes exhibit an iLOGP of less than 1 (Fig. 2E). Interestingly, the fragment-based miLogP descriptor showed 87% of permeant probes to have a miLogP equal or greater than 1 and 96% of impermeant probes exhibited lower miLogP values (Fig. 2F).

Overall, the fragment-based miLogP descriptor shows a good accuracy in correctly categorizing the permeability of permeant as well as impermeant probes. Nevertheless, it tends to underestimate the LogP for some probes of diverse chemistries (Table 1), which may increase the false negative rates^8^. This could also explain the wrong categorization of 13% of the permeant probes as to be impermeant.

**Table 1:** Comparison of miLogP and experimental partition coefficient values.

Probe name	miLogP	Partition Coeff	Reference
Bodipy (cpd_1)	− 2.47	3.08	^37^
Bis-pyridinium (cpd1a)	− 5.33	− 0.5	^38^
Bis-pyridinium (cpd1b)	− 3.57	− 0.4	^38^
Bis-pyridinium (cpd1c)	− 5.33	− 0.6	^38^
Bis-pyridinium (2)	− 4.04	0.1	^38^
Bis-pyridinium (3)	2.95	0.8	^38^

Partition Coeff. = Experimental Partition Coefficient.

### Deep Neural Network training and validation

**Figure 3:**
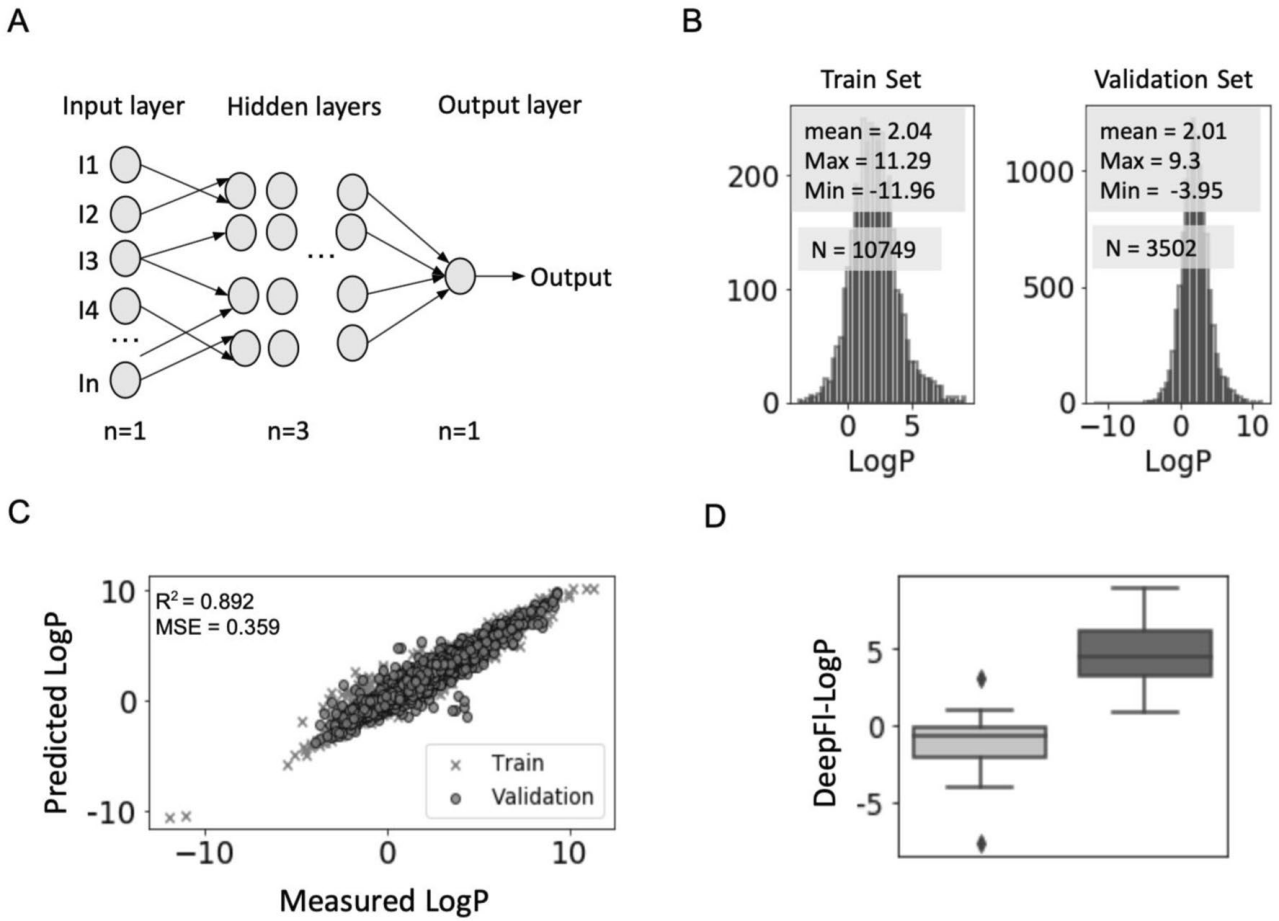
Deep neural network architecture and performance of the neural DeepFl-LogP descriptor. (**A**) Diagram of the deep neural network architecture. (**B**) Partition coefficient distribution of the training data set and the data set for validation. (**C**) Training/ validation of the DeepFl-LogP descriptor. (**D**) Distribution of the DeepFl-LogP descriptor for the test set consisting of impermeant probes (light grey) and cell permeable probes (dark grey). Please note that the average DeepFl-LogP value of permeant probes is significantly higher than that of impermeant probes (t-test, *p* value = 2.79e−14).

To address this limitation of the miLogP descriptor, a novel LogP algorithm based on a feedforward deep neural network (DNN) has been developed. The DNN has an input layer comprised of 319 neurons, which is sequentially connected to 3 hidden layers and an output layer of a single neuron was used (Fig. 3A). More information about the network architecture and its hyperparameters are included in the materials and methods section. For DNN training and validation, the publicly available OPERA dataset was used^30^. It contains high-quality training/validation sets, which were curated from the publicly available PHYSPROP ^34^ database. The training and the validation set used here consist of the experimental determined LogP values of more than 10,000 and 3,000 molecules, respectively. In order to cover a wider range of LogP values and to enlarge the reference chemical space, the original OPERA training set was enlarged by including additional molecules (n = 222) (Table S4). Twelve of those represent fluorescent derivatives (see material and methods). However, training and validation sets with comparable LogP characteristics (distribution) and a very close mean were maintained (Fig. 3B), which is crucial for an accurate estimation of the network’s performance.

The DNN was trained by calculating a total of 319 molecular fingerprints and fragments (Counts/Booleans) for each molecule. The molecular fingerprint is a map that represents the atom types and their bonding information of a particular molecule. In the training step, the E-State indices (bonding information) of the atoms were excluded. The sufficiency of the atom-types information and the expansion of the chemical space of the fragments search (i.e. substructure analysis, Table S3) for an accurate LogP prediction was hypothesized. The final trained model yielded a test R^2^ of 0.892 and a low mean square error (MSE) of 0.359 (Fig. 3C). The final DNN, including its weights, was saved for later use.

### DeepFL-LogP descriptor an accurate predictor of permeability

In order to validate the performance of the DNN algorithm in predicting the cell permeability of fluorescent probes, we have determined the DeepFl-LogP descriptor, which was immediately calculated for a test data set of fluorescent probes and fluorophores. The results show good agreement with previous permeability models^5,8,35^. The average DeepFl-LogP of the permeant probes is significantly higher than that of the impermeant probes (*p* value = 2.79e−14) (Fig. 3D). The majority (96%) of the permeant and the impermeant probes exhibited a LogP ≥ 1 or a LogP < 1, respectively.

**Figure 4:**
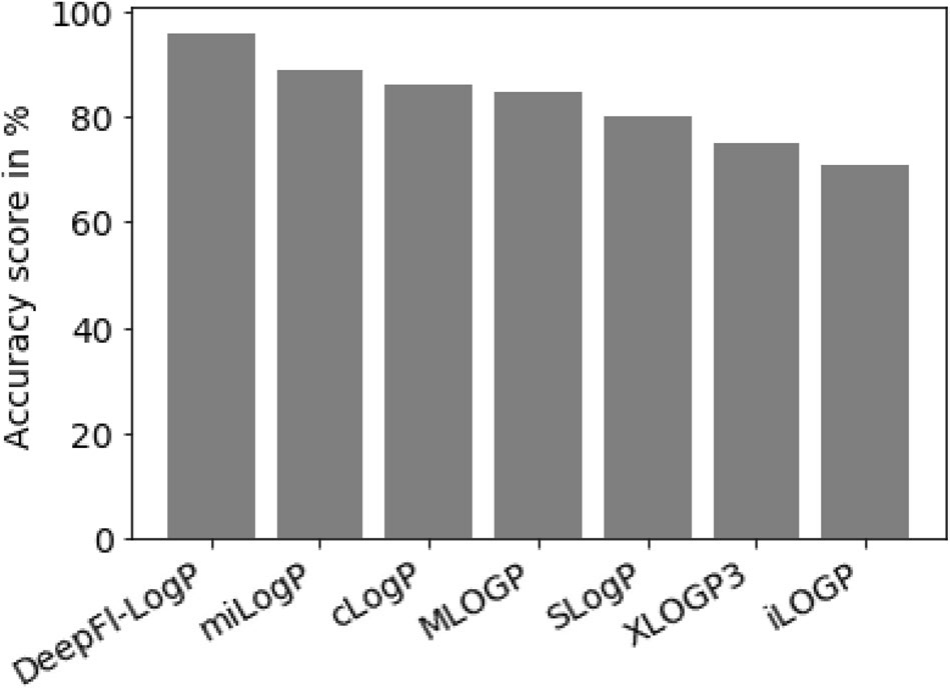
Ranking of LogP descriptors. The LogP descriptors are arranged in descending order according to accuracy score.

**Figure 5:**
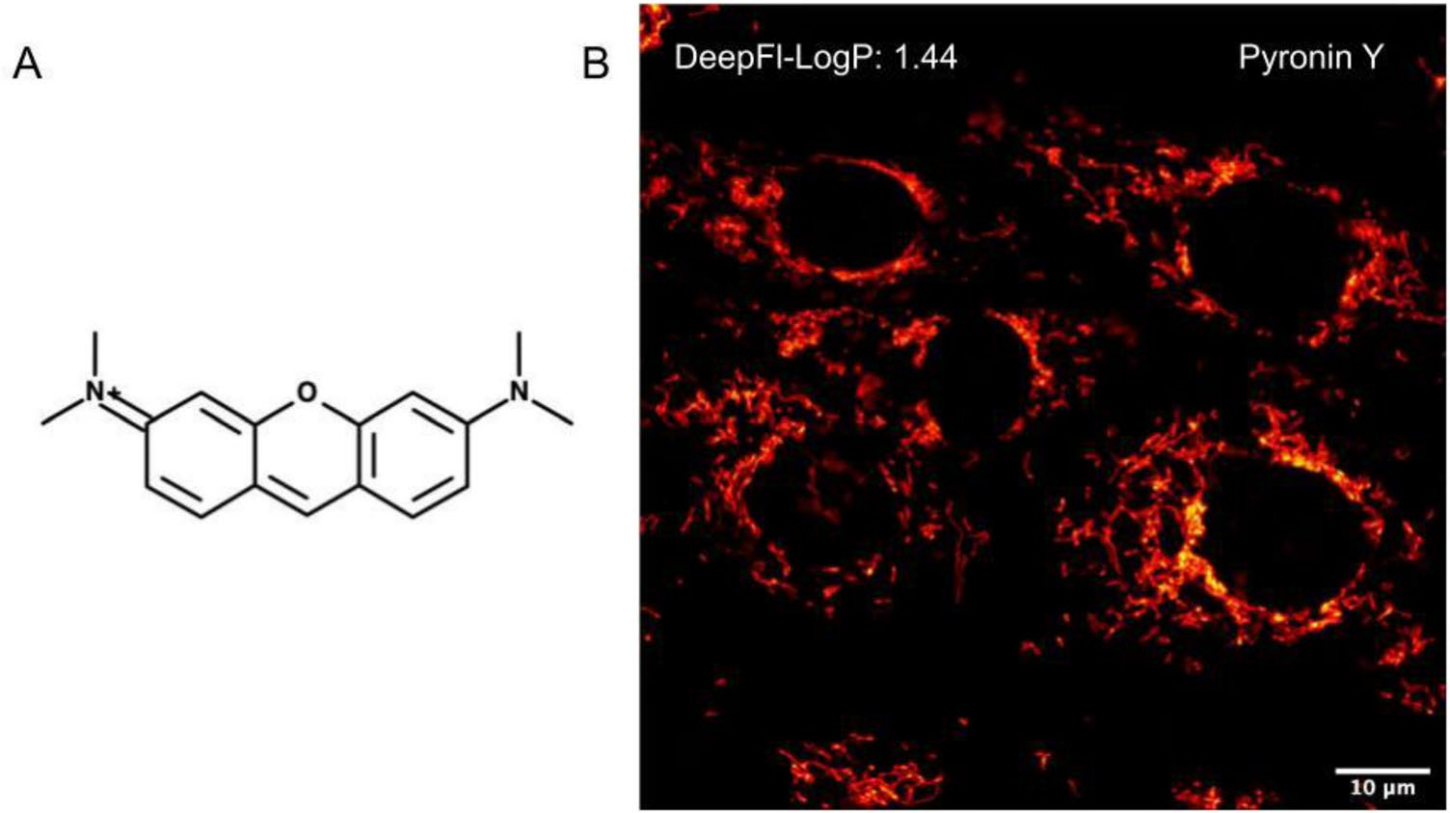
Pyronin Y confocal imaging of live cells. (**A**) Chemical structure of the Pyronin Y dye. (**B**) Confocal image of mitochondria in living Vero cells stained with the Pyronin Y (1 µM). Scale bar is 10 µm.

The previous analysis indicates that the fragment-based descriptors (miLogP/DeepFl-LogP) perform better in predicting the permeability of fluorescent chemical probes on basis of a simple LogP threshold model. To verify this presumption quantitatively, the accuracy for all the LogP descriptors was determined and compared utilizing the same test set of probes. Accuracy represents the number of correctly categorized probes. Ranking the descriptors according to the highest accuracy score shows that DeepFl-LogP and miLogP descriptors perform best among all other descriptors (Fig. 4). The DeepFl-LogP even outperforms the miLogP descriptor, as it has a higher accuracy score than that of the miLogP (96% vs 89%). This significant improvement in the accuracy has immediate practical implications, especially when low LogP values lead to higher false negative rates. For example, the well-known cell-permeant cationic dye (pyronin Y) could have been easily misclassified as an impermeant dye if the miLogP descriptor was used to judge its’ permeability (miLogP for Pyronin Y = − 0.01). This is not the case with the DeepFl-LogP descriptor (Fig. 5).

## Discussion

The development of new membrane-permeant fluorescent probes with predefined properties is highly important for applications in diagnostics and research. In a typical development process, improving the permeability of a new probe design is a tedious process which requires many cycles of optimization, in which the binding specificity must be maintained or even additionally optimized further. In the case of fluorescent probes for super-resolution imaging, additional requirements on the photophysical properties, such as photostability, also have to be met. All these properties are sensitive to subtle changes in the chemical structure. Therefore, the development of a robust descriptor or an equivalent QSPR model that can be used to accurately predict one or more of the aforementioned properties could help to speed up the development process and thus reduce the overall development costs.

In this study, we developed a simple model, based on thresholding a LogP descriptor, which can predict with good accuracy whether a fluorescent probe is cell permeable or not. Our analysis of different LogP descriptors showed that fragment based LogP descriptors exhibit the best accuracy in categorizing the permeability of fluorescent probes within this model. We also showed that this accuracy can be further improved by using a novel DNN based LogP descriptor.

As the development of new permeable fluorescent probes is often limited by the poor permeability and solubility of the precursor fluorophore, in silico approaches are ideally suited as a first screen for predicting permeation. Current LogP descriptors are not computationally expensive, but we showed in our study that they cause a large misclassification error when it comes to predicting the cell permeability of fluorescent probes. This is not the case for the DeepFl-LogP descriptor. It is fast (a few microseconds computing time per molecule) and can therefore easily be used as a permeability prediction tool for fluorescent compounds. Such a LogP-screening of probe designs prior to synthesis is certainly faster and cheaper than the hitherto existing workflow of iterative synthesis, purification and testing of each novel fluorescent compound.

Finally, we expect that our results as well as the newly developed DeepFl-LogP descriptor will also be beneficial for other types of in silico studies. For example, is LogP an important descriptor in most quantitative toxicity relationship (QSTR) models, which are used to assess the risks of chemical exposure^13,36^.

## Conclusion

Small-molecule fluorescent probes are becoming powerful biomedical reagents to advance cell biology and drug discovery research, as well as cancer diagnostics. The majority of applications are bioimaging applications and the design of these probes is usually a two-fold problem: The photophysical properties of the incorporated fluorophore has to be optimum, especially for super-resolution imaging applications, as well as the physicochemical properties, such as the probe permeability. The cell permeability of probes affects both the staining quality and toxicity of the applied molecules. In silico methods for predicting these properties are promising tools for the enhancement of the development of molecules with favorable properties. Nevertheless current permeability models are based on multi-descriptors and statistical models, yet they predict the permeability of fluorescent probes with moderate accuracy. In praxis, to rely on the available tools with moderate accuracy can be counterproductive, especially when searching a wide range of chemical space and at the same time being limited in chemical resources.

LogP has been a key molecular descriptor in predicting the cell permeability of molecules. Here, we tested if a simple permeability model that is solely based on this descriptor can accurately predict the cell permeability of complex fluorescent molecules. By screening several standard LogP algorithms, we found that the fragment-based LogP algorithms exhibit a high accuracy in categorizing the permeability of structurally diverse fluorescent probes. Further, we developed an improved deep neural fragment-based LogP descriptor (DeepFl-LogP). The training set of the neural network included additional molecules to those found in the OPERA database. Increasing the reference chemical space and the use of a larger molecular fingerprint in the modeling step enabled us to substantially improve the overall accuracy of the DeepFl-LogP, and more important to categorize permeability of chemically diverse fluorescent probes.

DeepFl-LogP is the first tool, which can be used with confidence as a predictor for one of the most important properties when it comes to the development of cell permeable organic probes. In order to make the DeepFl-LogP tool publicly available, the Python script and the datasets are in our GitHub repository (www.github.com/k-soliman/DeepFl-LogP).

## Supplementary Information

Supplementary Information 1.

Supplementary Information 2.

## Data Availability

All the datasets and the code are available at www.github.com/k-soliman/DeepFl-LogP.
