# Supplementary material for: Predicting the membrane permeability of organic fluorescent probes by the deep neural network based lipophilicity descriptor DeepFl-LogP
Source: Sci Rep. 2021 Mar 26;11:6991. doi: 10.1038/s41598-021-86460-3 (PMC7997998; doi:10.1038/s41598-021-86460-3)

**Predicting the membrane permeability of organic fluorescent probes by the deep neural network based lipophilicity descriptor DeepFl-LogP**

Kareem Soliman^1^, Florian Grimm^2^, Christian A. Wurm^2^, Alexander Egner^1, 3^

**Affiliation**

^1^ Institute for Nanophotonics Göttingen e.V., Optical Nanoscopy, Hans-Adolf-Krebs Weg 1, 37077, Göttingen, Germany

^2^ Abberior GmbH, Hans-Adolf-Krebs Weg 1, 37077, Göttingen, Germany

^3^ George-August University, Faculty of Physics, Göttingen, Germany

**E-Mail:** [kareemsoly@yahoo.com](mailto:kareemsoly@yahoo.com)

**Figure S1. Fluorescent compounds representation in the original OPERA training dataset (blue), DeepFl-LogP Training dataset (Red), and the Test dataset of fluorescent probes.** The compounds were arranged according to the class of the fluorophore.


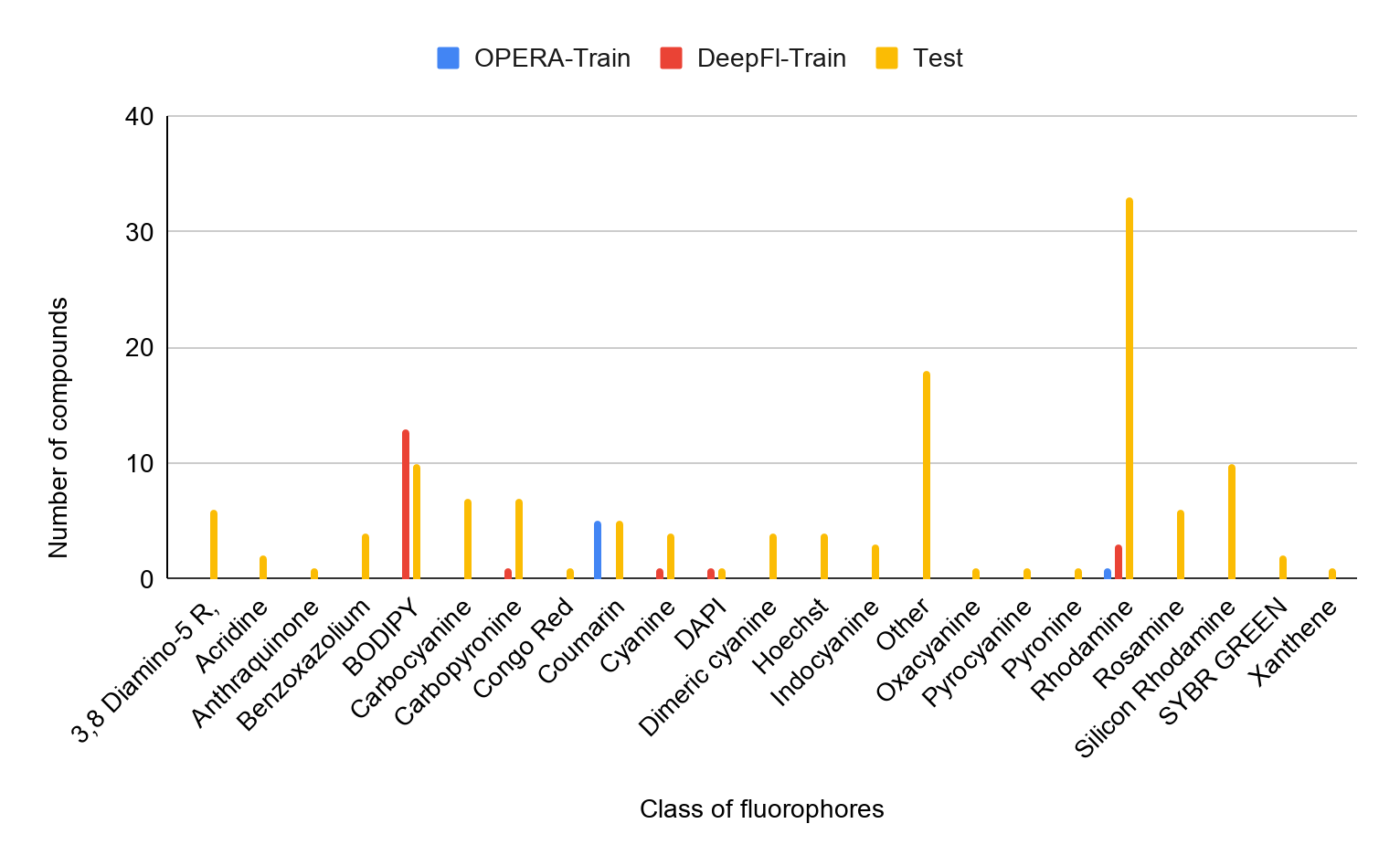

Supplement: Supplementary file 2 — Supplementary Information 2. [file 41598_2021_86460_MOESM2_ESM.docx]
